# Heme Induction with Delta-Aminolevulinic Acid Stimulates an Increase in Water and Electrolyte Excretion

**DOI:** 10.1155/2012/690973

**Published:** 2012-01-23

**Authors:** Syed Quadri, Debra W. Jackson, Priyanka Prathipati, Courtney Dean, Keith E. Jackson

**Affiliations:** ^1^Department of Basic Pharmaceutical Sciences, College of Pharmacy, The University of Louisiana at Monroe, Monroe, LA 71201, USA; ^2^Department of Biology, College of Arts and Sciences, The University of Louisiana at Monroe, Monroe, LA 71209, USA; ^3^Department of Physiology and Tulane Hypertension and Renal Center of Excellence, Tulane University School of Medicine, New Orleans, LA 70112, USA

## Abstract

*Purpose*. Studies were performed to examine hemodynamic and renal function before and after acute induction of the endogenous CO system with delta-aminolevulinic acid (DALA), which drives HO activity. *Methods*. *In vivo* studies were conducted on Inactin-anesthetized male Sprague Dawley rats (250–300 g) either with or without chronic pretreatment with L-NAME (50 mg/Kg, q12 hours x4d). *Results*. DALA (80 *μ*mol/Kg, IV bolus) administration acutely increased endogenous CO production and HO-1 protein. In untreated and L-NAME-pretreated rats, DALA did not alter BP, GFR, or RBF but increased UF, U_Na_V, and U_K_V (untreated: Δ108.8 ± 0.28%, 172.1 ± 18.4%, and 165.2 ± 45.9%; pretreated: Δ109.4 ± 0.29%, 187.3 ± 26.9%, and 197.2 ± 45.7%). Acute administration of biliverdin (20 mg/kg, IV) and bilirubin (30 mg/kg, IV) to similarly treated animals did not alter UF, U_Na_V, and U_K_V. *Conclusion*. These results demonstrate that heme oxygenase induction increases urine and electrolyte excretion and suggest a direct tubular action of endogenous carbon monoxide.

## 1. Introduction

Metabolic degradation of heme by heme oxygenase (HO) yields three products; biliverdin, ferrous iron, and carbon monoxide (CO) [1]. Currently, two major isoforms of the HO enzyme have been recognized, the inducible HO-1 and the constitutive HO-2. Both isoforms have been reported to be present in the kidneys [1–3]. Several biological stressors, such as oxidative stress, ischemia, and hypertension, are known to increase HO-1 levels [4–6]. In contrast, the HO-2 isoform is constitutively expressed and is present in high concentrations in the kidney, as well as in other vascular beds and tissues [7]. Alterations in HO levels have been demonstrated to alter CO concentration, in addition to having profound effects on vascular tone [8, 9].

Current literature supports both an endothelial-dependent vasoconstrictor effect of CO and an endothelial-independent vasorelaxation [10, 11]. CO-mediated vasoconstriction is via inhibition of nitric oxide synthase (NOS) [11, 12]. CO also promotes endothelium-independent vasodilation through the activation of soluble guanylyl cyclase, stimulation of K channels, and inhibition of the cytochrome-P450-dependent monooxygenase system in vascular smooth muscle cells [10, 13]. Increases in endogenous CO levels produce a decrease in blood pressure in several forms of hypertension, while HO inhibition increases arterial blood pressure [4, 14–17]. Regional differences in renal blood flow (RBF) have been demonstrated with increases in the medulla without significant increases in cortical blood flow during heme-induced increases in CO [13]. Other studies have not shown significant alterations in renal vascular resistance during alterations in CO levels, thus controversy does exist in the literature as it relates to the ability of CO to regulate renal vascular resistance [18].

Increases in HO activity via heme administration promote vasorelaxation and produce diuresis and natriuresis [19]. In addition, several studies have identified an antioxidant role for bilirubin and biliverdin during stress [20, 21]. However, the mechanisms of HO-mediated effects on renal function have yet to be elucidated. Because the HO-mediated diuretic and natriuretic effects were observed concomitantly with an increase in RBF, it is possible that alterations in renal hemodynamics mediate the increase in UF and sodium excretion. It has also been reported that renal medullary HO plays a key role in the regulation of pressure natriuresis and, thus, the control of arterial blood pressure [22]. Macula densa cells have been reported to express HO-1 and HO-2, and stannous mesoporphyrin, an inhibitor of HO, was shown to augment tubuloglomerular feedback in both *in vitro* and *in vivo* studies [23]. In addition, we recently reported that CO inhibition promotes antidiuresis and antinatriuresis independent of vascular or systemic changes [24]. Therefore, we hypothesized that increased levels of endogenous CO promote natriuresis and diuresis independent of inhibition of nitric oxide synthase (NOS) and alterations in RBF. To examine this hypothesis, the potential direct tubular effects of a heme precursor, delta-aminolevulinic acid (DALA), which drives HO activity, were studied using a dose of DALA that does not elicit changes in renal hemodynamic function in control and L-NAME treated rats.

## 2. Methods

### 2.1. Materials

DALA was purchased from Frontier Scientific (Logan, UT, USA). Inactin (thiobutabarbital sodium), N-Nitro-L-Arginine Methyl Ester (L-NAME), bilirubin, and para-aminohippuric acid (PAH) were obtained from Sigma-Aldrich (St. Louis, MO, USA). Albumin was purchased from EMD Biosciences Inc. (San Diego, CA, USA). Inulin was purchased from Fresenius Kabi UK Ltd. (Runcorn, Cheshire). Plasma Renin Activity (PRA) kits were purchased from Diasorin Inc. (Stillwater, MN, USA). Biliverdin was purchased from MP Biochemicals, LLC (Solon, OH, USA). All other chemicals were purchased from Fisher Scientific (Houston, TX, USA). DALA stock solution (800 mmol/L) was prepared in saline on the day of the experiments. L-NAME (50 mmol/L) was dissolved in saline immediately before intraperitoneal injection. All other solutions were freshly prepared on the day of the experiment.

### 2.2. Animals

Male Sprague-Dawley rats (250–350 g; *n* = 146, Harlan, Indianapolis, IN, USA) were used (*n* = 32). This protocol was approved by the Tulane School of Medicine and University of Louisiana at Monroe Institutional Animal Care and Use Committee. Prior to experiments, rats were housed in a controlled environment and had free access to commercial rat chow and tap water. Subsets of animals were chronically treated every 12 hours for four days with an inhibitor of NOS [25], L-NAME (50 mg/kg, IP). To minimize postprandial sodium excretion variability, animals were deprived of food for 12 hours before experiments.

Subsets of animals were chronically treated with L-NAME every 12 hours for four days. After anesthetization with Inactin and surgical preparation, rats were allowed to stabilize for 45 min. After this initial stabilization period, a 30-minute control period was performed and urine was collected. L-NAME-treated and -untreated animals were then acutely administered DALA (80 *μ*mol/kg, IV), biliverdin (20 mg/kg, IV), bilirubin (30 mg/kg, IV), or vehicle (1 mL saline, IV), and an additional 30-minute treatment period was performed. The doses of biliverdin and bilirubin were chosen from previous studies where an antioxidant effect was observed [20, 21]. Mean arterial pressures (MAP), heart rates (HR), and RBF were measured during both the 30-minute control and treatment periods. After the experimental protocols were completed, renal vascular resistance (RVR) was calculated as the pressure to flow ratio and expressed as “mmHg/(mL/min)”.

### 2.3. Experimental Procedures

Rats were anesthetized with a single injection of thiobutabarbital sodium (120 mg/kg; IP), and a tracheal tube was inserted to maintain an open airway. Fluid filled catheters (PE-50 tubing filled with heparinized saline) were inserted into a carotid artery and a jugular vein to allow for continuous monitoring of MAP and HR, and for intravenous administration of drugs, respectively. The arterial catheter was connected to a pressure transducer (model TSD104A, Biopac Systems, Santa Barbara, CA, USA), and the venous catheter was connected to a Sage microinfusion pump (Orion Research, Inc., model M361, Boston, MA, USA) set at 1 mL/hr saline infusion rate. A bladder cannula was inserted to allow urine collection for determination of urine flow and concentrations of sodium and potassium (Flame Photometry; Instrumentation Laboratories, IL 943). A flank incision was made to expose the left kidney and renal artery. RBF was measured with a renal flow probe (Transonic, Ithaca, NY, USA) placed around the renal artery and connected to a Transonic-T206 synchronized flow meter coupled to a polygraph system (model MP100, Biopac System).

### 2.4. Glomerular Filtration Rate

In a subset (*n* = 24) of anesthetized rats, the experiments were repeated with an additional catheter inserted into the right femoral vein to infuse inulin, para-amino hippuric acid, and albumin. Plasma and urine sodium and potassium concentrations were determined by flame photometry, and inulin concentrations were measured colorimetrically to determine glomerular filtration rate (GFR) [26]. RVR and fractional sodium excretion (FE_Na_) were calculated according to standard formulas. The renal excretion data from this subset of animals were not included in the final measures due to the different handling of these animals (additional catheter, and albumin, PAH, and inulin infusion). However, the excretory data from these animals followed the same trends as the reported data.

### 2.5. Plasma Renin Activity

Plasma renin activity (PRA) was measured with a commercially available assay kit (Gamma Coat PRA Assay Kit) [27]. Briefly, DALA (80 *μ*mol/kg; IP) was infused into L-NAME- (50 mg/Kg; IP) pretreated or untreated rats and PRA was measured to determine if altered CO levels had any effect on the renin-angiotensin system. PRA was determined by the radioimmunoassay generation of angiotensin I. Given the noted experimental difficulties with measuring PRA in whole animals, we did not perform clearance measurements or CO measurements in these animals.

### 2.6. Determination of the Effect of DALA to Increase CO Excretion

A subset of awake Sprague-Dawley rats (*n* = 12) that did not receive any surgical treatments were infused with DALA (80 *μ*mol/kg, IV) to increase HO activity, both with and without chronic L-NAME pretreatment every 12 hours for 4 days. Animals were placed in an acrylic airtight chamber with the outflow leading to a heated mercuric oxide bed coupled with a gas chromatograph (Peak, Mountain View, CA, USA) for the determination of CO concentration, detailed elsewhere [28, 29]. The chamber was continuously purged with purified air and the outflow sampled for CO concentration at 2 min intervals. After a 10 min equilibration period, the average of four measurements was used to calculate the CO excretion rate for the whole animal.

### 2.7. Determination of Renal HO-1 Levels

In a subset (*n* = 14) of similarly treated anesthetized animals, the experimental protocols were repeated to determine the ability of DALA infusion to alter renal HO-1 levels. Renal HO-1 levels were measured by commercially available ELISA kits purchased from Stressgen. Kidneys from L-NAME-pretreated and untreated rats were removed and flash frozen in liquid nitrogen and suspended in 1X extraction reagent and protease inhibitor. Once the kidney tissues were homogenized, the ELISA sandwich immunoassay was preformed and the level of HO-1 protein present in the kidney was determined.

## 3. Data Analysis

Data were expressed as mean ± SEM. Data were analyzed by analysis of variance (ANOVA) followed by orthogonal contrast when appropriate (SYSTAT). Bonferroni correction was employed in the final analysis of completed series (*α* = 0.05) [30].

## 4. Results

### 4.1. Whole Animal CO Excretion

**Figure 1:**
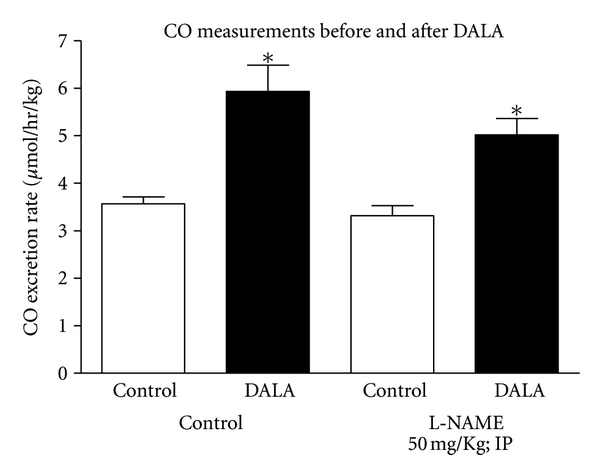
DALA (80 *μ*mol/kg; IV) infusion acutely increased expired CO levels in L-NAME-treated and -untreated awake rats. Values are mean ± SE; *n* = 6 each.

Acute administration of the heme precursor, DALA (80 *μ*mol/kg, IP), to untreated animals produced a significant increase in expired CO levels (Δ63.9 ± 1.6%, *n* = 3) (Figure 1). This effect was similar to a higher dose of DALA (800 *μ*mol/kg, IP) (Jackson et al, unpublished results). This increase in expired CO was not affected by L-NAME (50 mg/Kg; IP) pretreatment (Δ67.6 ± 1.9%, *n* = 3) (Figure 1).

### 4.2. Renal HO-1 Levels

**Figure 2:**
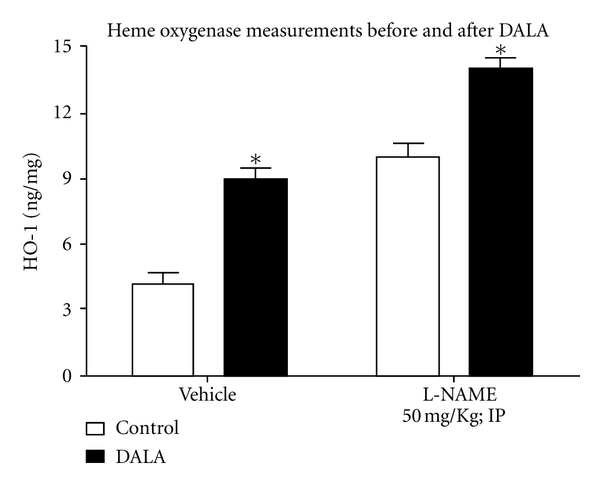
In anesthetized rats, DALA (80 *μ*mol/kg; IV) acutely increased renal HO-1 levels in vehicle (left) and L-NAME-treated (right) rats. (*P < 0.05, pre- versus 30 min post-DALA; *n* = 6 each).

Acute administration of DALA (80 *μ*mol/kg, IV) in untreated and L-NAME-pretreated anesthetized rats produced a significant increase in renal HO-1 levels in untreated (Δ50 ± 0.56%,  *n* = 7) (Figure 2) and L-NAME-treated (Δ60 ± 0.64%,  *n* = 7) (Figure 2) rats. L-NAME pretreatment produced a significant increase in renal HO-1 levels, as compared to untreated animals (Figure 2); however, DALA increased renal HO-1 levels to a similar extent as in untreated animals. There were no significant differences in hematocrit pre- and post-DALA administration in both the L-NAME-pretreated and -untreated animals. 

### 4.3. Renal Functional Responses

**Figure 3:**
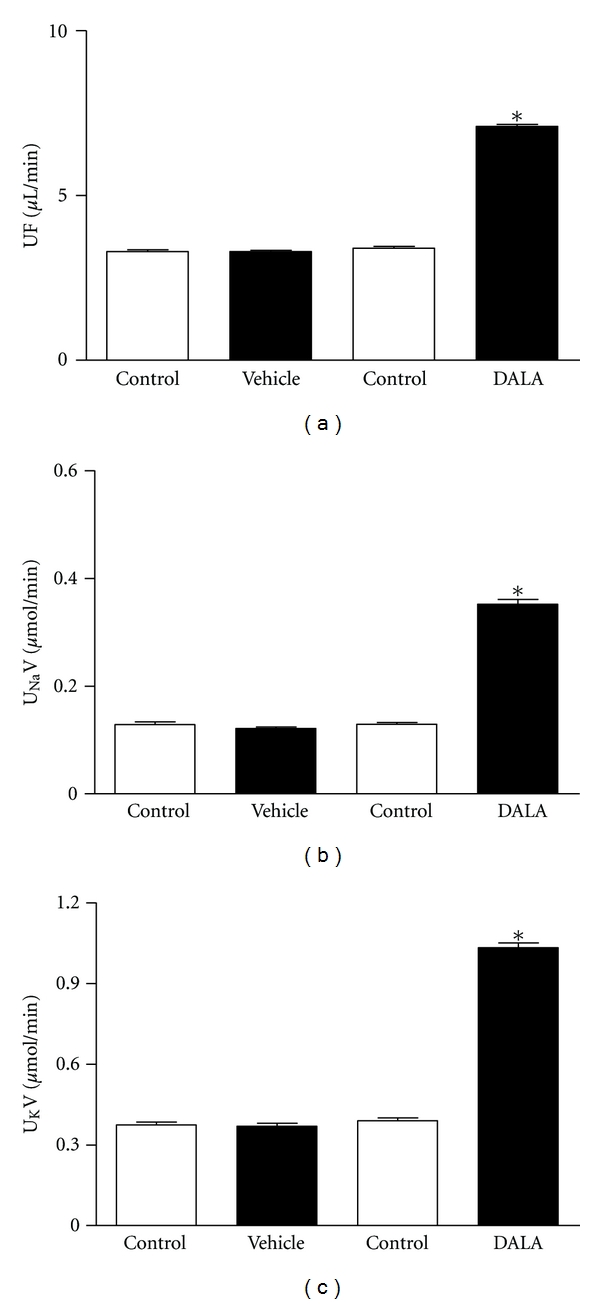
In anesthetized rats, DALA (80 *μ*mol/kg) IV infusion acutely increased urine flow and sodium and potassium excretion in untreated rats. Values are mean ± SE; *n* = 20.  *P < 0.05 versus control (vehicle infusion).

**Figure 4:**
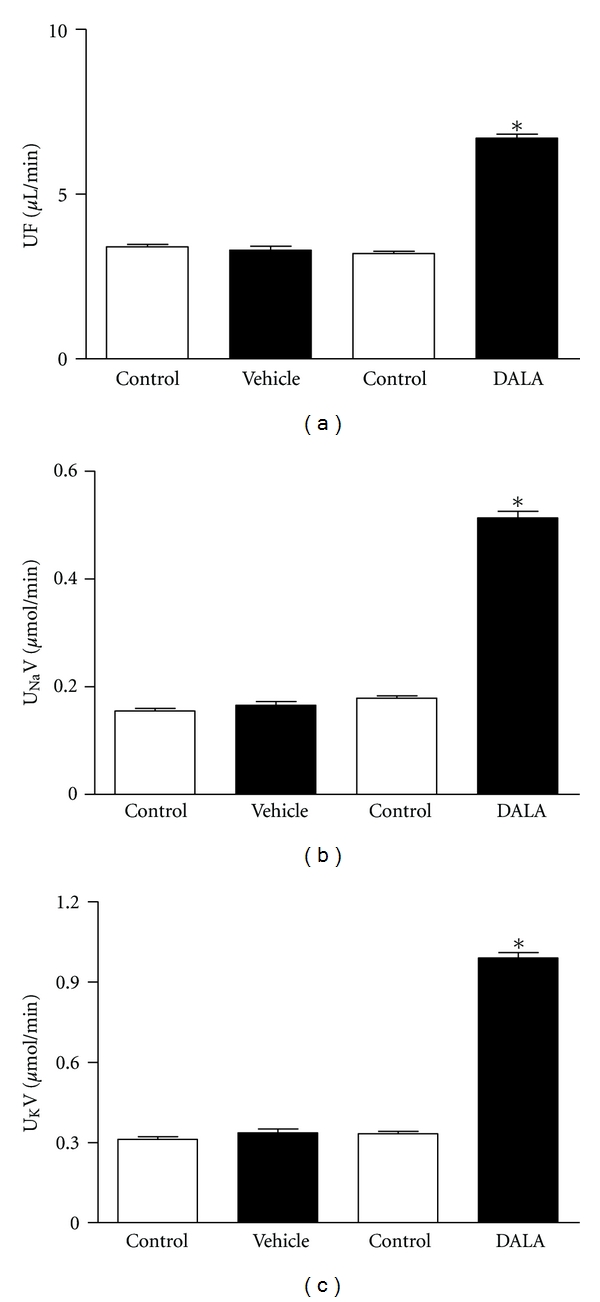
In anesthetized rats, acute IV infusion of DALA (80 *μ*mol/kg) increased urine flow, urinary sodium, and urinary potassium excretion in L-NAME-pretreated animals. Values are mean ± SE; *n* = 20.   *P < 0.05 versus control (vehicle infusion).

The subsequent values were obtained during the 30 min experimental period following administration of DALA (80 *μ*mol/kg; IP), biliverdin (20 mg/kg), bilirubin (30 mg/kg), or vehicle in L-NAME-(50 mg/kg; IP) treated and untreated animals. In animals without pretreatment, DALA did not exert significant systemic or renal hemodynamic effects (Table 1), but there were significant increases in urine flow and sodium and potassium excretion (Δ108.8 ± 0.28%, 172.1 ± 18.4%, and 165.2 ± 45.9%: *n* = 20) (Figure 3). Biliverdin (20 mg/kg) and bilirubin (30 mg/kg) did not cause significant systemic or renal hemodynamic effects (Table 1) and any significant changes in urine flow or sodium, and potassium excretion (Table 3). In rats pretreated chronically with L-NAME, there was a significant increase in MAP (100 mmHg versus 150 mmHg) but DALA administration had no significant effects on MAP, HR, RBF, or RVR (Table 1). However, DALA significantly increased urine flow and sodium and potassium excretion (Δ 109.4 ± 0.29%, 187.3 ± 26.9%, and 197.2 ± 45.7%: *n* = 20) (Figure 4). Biliverdin (20 mg/kg) and bilirubin (30 mg/kg) did not exert significant effects on MAP, HR, RBF, or RVR (Table 1) and any significant effects on urinary volume, sodium and potassium excretion (Table 3). There were no significant differences between the urine flow and electrolyte excretion in the L-NAME-untreated and -treated animals. Vehicle treatment had no effect in either group. DALA had no effects on glomerular filtration in either L-NAME-treated or -untreated rats (Table 2: *n* = 24).

### 4.4. Plasma Renin Activity

**Figure 5:**
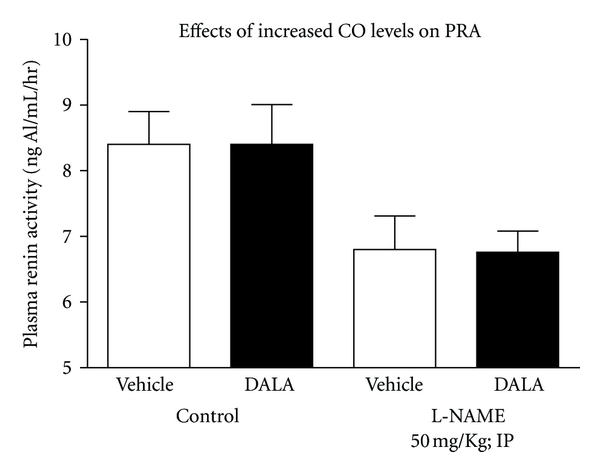
Acute administration of DALA (80 *μ*mol/kg; IP) did not exert significant effects on plasma renin activity (PRA) in L-NAME-pretreated and -untreated rats. Vehicle and DALA changes in PRA were observed for 30-minute periods. Values are mean ± SE; *n* = 46.

In untreated rats given DALA, no significant differences in plasma renin activity (PRA) were evident (Figure 5; *n* = 25). Similarly DALA did not significantly alter PRA in L-NAME-pretreated animals (Figure 5; *n* = 21).

## 5. Discussion

The present study investigated the role of increases in endogenous CO on renal excretory function. The heme precursor, DALA, increased expired CO levels in both L-NAME-treated and -untreated animals. DALA, which promotes the generation of endogenous CO, increased volume and electrolyte excretion in both L-NAME-treated and -untreated animals. Acute increases in endogenous CO formation were not accompanied by any significant differences in systemic or renal hemodynamic function in that a low dose of DALA was specifically chosen to avoid alterations in renal or systemic hemodynamics. There were also no significant changes in GFR with DALA infusion in L-NAME-treated or -untreated animals.

 Increases in heme oxygenase activity, promote an equimolar elevation in carbon monoxide, iron, and biliverdin [31]. Biliverdin is quickly converted to bilirubin [31]. Current literature would support an antioxidant role for both biliverdin and bilirubin [20, 21]. To examine the potential role of these heme products to alter renal excretory function, a subset of animals was given biliverdin or bilirubin and the study was repeated. However, no significant differences in renal or systemic hemodynamics were observed and, similarly, no significant differences in renal excretory function were observed, thus, suggesting that the observed increases in urine flow and sodium and potassium excretion were due to carbon monoxide. The negative results observed with biliverdin and bilirubin suggest that they are not involved in the heme-oxygenase-mediated diuretic effects; however, the current study cannot rule out the importance of these metabolites in the kidney in that renal intracellular concentrations of biliverdin and bilirubin were not measured.

DALA has been previously shown to increase HO activity in rats [32]. We have reported that DALA also increases expired CO levels, as well. Thus, DALA, a substrate that drives heme formation and increases HO activity, can produce significant increases in endogenous CO formation. DALA administration was observed to significantly increase HO-1 levels both in the presence and absence of an intact NO system. L-NAME administration increased baseline HO-1 levels, perhaps due to the observed elevation in MAP. Importantly, the ability of DALA administration to increase HO-1 levels was not affected by L-NAME.

The current study used DALA to drive CO formation in that iron loading can lead to effects on the vasculature that are independent of the CO system. Iron loading can occur, when one increases CO formation via heme administration or CO releasing molecules [32]. The current data support the hypothesis that CO increases water and electrolyte excretion independent of changes in systemic or renal hemodynamics. The increases in urine flow and electrolyte excretion were still present during NOS inhibition by L-NAME, indicating once again that the CO effects on urine flow and electrolyte excretion are not simply due to alterations in the nitric oxide (NO) system. Furthermore, DALA administration did not significantly alter PRA, thus CO enhancement of renal excretion was not via suppression of the renin angiotensin system. We recently reported that inhibition of endogenous CO increased PRA in untreated rats [24]. This increase in PRA was abolished by L-NAME pretreatment [24]. However, endogenous CO effects on the juxtaglomerular cells could be maximal even at basal conditions; therefore, increasing CO formation by DALA would not affect PRA.

In previous research, an increase in CO concentration elicited through heme administration was shown to decrease RVR, increase RBF, and urine flow and sodium excretion [33]. Similar results can be observed with CO releasing molecules. Pretreatment with the HO inhibitor, SnMP, abolished the diuretic and natriuretic effects of heme but did not affect the increases in RBF. The heme-induced changes in renal hemodynamic parameters could perhaps be attributed to differences in agents (DALA versus heme) and/or concentrations. Regional differences in HO activity in the kidney have been reported, where medullary heme oxygenase contributes to pressure natriuresis and arterial blood pressure in the absence of any significant changes in cortical HO activity [22]. As previously stated, low concentrations of DALA were employed to avoid hemodynamic changes in the present study. Therefore, DALA-induced increases in urine flow and electrolyte excretion were not accompanied by any changes in renal hemodynamic function. However, medullary blood flow was not measured and we cannot exclude the possibility of small increases in medullary BF to the diuretic and natriuretic responses. Collectively these data suggest that CO alters water and electrolyte excretion independent of changes in NO and renal hemodynamic function and suggests that this response is due to a direct renal tubular effect.

Previous studies have demonstrated CO's ability to promote vasoconstriction via inhibition of NOS [19, 34]. However, in the present study, such an interaction between the two systems in acutely regulating water and electrolyte excretion was not observed. Thus, it is possible that in organ systems with a large capacity to autoregulate, such as the brain, heart, and kidney, CO inhibition of NO does not play a major role in establishing normal basal vascular tone. CO was able to promote water and electrolyte excretion without affecting renal hemodynamics, which suggests an alternate pathway for CO regulation of renal excretory function. Thus, CO could have direct effects on the tubules to alter water and electrolyte excretion. As a low dose of DALA was administered to avoid altering renal hemodynamics, the results suggest that the alterations in renal excretory function are most likely mediated via a direct tubular effect to inhibit sodium transport in that sodium and potassium excretion were enhanced during DALA administration.

## 6. Significance of the Study

Previous studies have demonstrated that increases in HO activity can promote significant diuresis [33]. Since heme administration was accompanied by a significant increase in blood pressure, it could not be established if the observed diuresis was due to a direct tubular action or simply due to an increase in perfusion pressure. In addition, it was not shown if the diuresis was due to CO or one of the other HO metabolites. In the current study, we demonstrate a direct tubular action of HO induction in the absence of alterations in renal hemodynamic function. Furthermore, the negative results with biliverdin and bilirubin administration suggest a tubular role of CO as a novel diuretic and therapeutic target to treat hypertension.

## 7. Conclusion

In summary, the present data indicate that an induction in HO-1 increases water and electrolyte excretion in the absence of alterations in renal hemodynamics, PRA, GFR, or NO production, thus, suggesting a direct tubular role for endogenous CO in the control of sodium excretion.

## Figures and Tables

**Table 1:** Effects of DALA (80 *μ*mol/kg, IV), biliverdin (20 mg/Kg, IV), and bilirubin (30 mg/Kg, IV) administration on heart rate (HR), mean arterial pressure (MAP), renal blood flow (RBF), and calculated renal vascular resistance (RVR).

		HR	MAP	RBF	RVR
	*N*	(bpm)	(mmHg)	(ml/min)	(mmHg/ml/min)
No pretreatment					
Control		385 ± 0.13	110 ± 0.06	5.6 ± 0.12	19.6 ± 0.14
Vehicle	8	395 ± 0.16	114 ± 0.08	5.8 ± 0.14	19.7 ± 0.11
DALA	8	382 ± 0.12	118 ± 0.09	5.9 ± 0.16	20.0 ± 0.18
Biliverdin	8	396 ± 0.25	113 ± 0.04	6.1 ± 0.15	18.6 ± 0.21
Bilirubin	8	398 ± 0.18	109 ± 0.15	5.4 ± 0.14	20.1 ± 0.24

Chronic L-NAME					
Control		398 ± 0.19	153 ± 0.05	5.5 ± 0.14	27.8 ± 0.28
Vehicle	8	400 ± 0.23	150 ± 0.12	5.2 ± 0.18	28.8 ± 0.20
DALA	8	403 ± 0.14	155 ± 0.24	5.8 ± 0.21	28.2 ± 0.38
Biliverdin	8	396 ± 0.19	158 ± 0.15	5.5 ± 0.14	28.7 ± 0.47
Bilirubin	8	399 ± 0.24	152 ± 0.05	5.1 ± 0.14	29.2 ± 0.32

**Table 2:** Effects of increases in endogenous CO (DALA 80 *μ*mol/Kg, IV) on glomerular filtration rate (GFR), urine flow (UF), sodium excretion (U_Na_V), fractional excretion of sodium (FE_Na_), and urinary potassium (U_K_V).

		UF	GFR	U_Na_V	FE_Na_	U_K_V
	*N*	(*μ*l/min)	(ml/min)	(*μ*mol/min)	(%)	(*μ*mol/min)
No pretreatment						
Control	6	6.49 ± 0.47	1.11 ± 0.05	0.59 ± 0.15	0.50 ± 0.03	0.14 ± 0.04
Vehicle	6	6.49 ± 0.48	1.10 ± 0.08	0.61 ± 0.08	0.51 ± 0.16	0.15 ± 0.03
Control	6	6.51 ± 0.50	1.12 ± 0.11	0.60 ± 0.11	0.53 ± 0.12	0.14 ± 0.01
DALA	6	13.99 ± 1.84*	1.10 ± 0.06	1.19 ± 0.03*	0.78 ± 0.01*	0.90 ± 0.13*

Chronic L-NAME						
Control	6	7.37 ± 0.73	1.11 ± 0.02	0.68 ± 0.13	0.50 ± 0.01	0.23 ± 0.13
Vehicle	6	7.43 ± 0.78	1.15 ± 0.18	0.68 ± 0.04	0.49 ± 0.05	0.23 ± 0.02
Control	6	7.51 ± 0.54	1.12 ± 0.17	0.67 ± 0.11	0.50 ± 0.02	0.24 ± 0.04
DALA	6	14.37 ± 0.41*	1.11 ± 0.10	1.22 ± 0.02*	0.80 ± 0.12*	1.09 ± 0.19*

**Table 3:** Effects of biliverdin (20 mg/Kg, IV) and bilirubin (30 mg/Kg, IV) administration on urine flow (UF), sodium excretion (U_Na_V) and urinary potassium (U_K_V).

		UF	U_Na_V	U_K_V
	*N*	(*μ*l/min)	(*μ*mol/min)	(*μ*mol/min)
No pretreatment				
Control	6	2.50 ± 0.30	0.17 ± 0.02	0.49 ± 0.06
Biliverdin	6	2.10 ± 0.12	0.16 ± 0.01	0.46 ± 0.05
Control	6	2.80 ± 0.15	0.19 ± 0.12	0.46 ± 0.01
Bilirubin	6	2.90 ± 0.21	0.16 ± 0.04	0.41 ± 0.09

Chronic L-NAME				
Control	6	3.40 ± 0.25	0.15 ± 0.02	0.41 ± 0.06
Biliverdin	6	3.30 ± 0.39	0.17 ± 0.02	0.40 ± 0.35
Control	6	3.20 ± 0.51	0.18 ± 0.02	0.46 ± 0.05
Bilirubin	6	3.30 ± 0.37	0.18 ± 0.02	0.45 ± 0.03
